# Crosstalk Between Leptin and Adiponectin in Colorectal Cancer: Molecular Mechanisms and Oncogenic Pathways

**DOI:** 10.3390/ijms27041789

**Published:** 2026-02-13

**Authors:** Svetla Slavova, Yoana Kiselova-Kaneva, Diana Ivanova, Deyana Vankova

**Affiliations:** 1 Department of Biology, Faculty of Pharmacy, Medical University “Prof. Dr. Paraskev Stoyanov”, 9000 Varna, Bulgaria; svetla.slavova@mu-varna.bg; 2 Department of Biochemistry, Molecular Medicine and Nutrigenomics, Faculty of Pharmacy, Medical University “Prof. Dr. Paraskev Stoyanov”, 9000 Varna, Bulgaria; yoana.kiselova@mu-varna.bg (Y.K.-K.); divanova@mu-varna.bg (D.I.)

**Keywords:** adipokine signaling, leptin, adiponectin, colorectal malignancies, obesity

## Abstract

Colorectal cancer (CRC) remains one of the most common malignancies worldwide with relatively high levels of morbidity and mortality. Current data demonstrate the significant role of adipokines, in particular leptin and adiponectin, in CRC pathogenesis and progression. Both adipokines exert pleiotropic activities and often possess opposing physiological effects. The main goal of this study was to provide a comprehensive overview of current knowledge regarding the complex relationship between leptin and adiponectin signaling and tumorigenesis with a specific focus on CRC. The pro-tumorigenic role of leptin in CRC has been highly emphasized by recent reports, primarily by activation of JAK2/STAT3 and PI3K/Akt/mTOR signaling pathways. In contrast, adiponectin has been shown to demonstrate an anti-tumorigenic role mainly because of activation of AMPK and PPARα signaling cascades. Focusing on the current advances in the field of adipokines’ signaling, we highlighted the latest achievements in understanding their role in colorectal malignancy.

## 1. Introduction

Colorectal cancer (CRC) is one of the most common malignancies, characterized by its high incidence and mortality rates. It is the third-most prevalent cancer in the world; however, it ranks second in cancer-related death [1]. In the recent past, it was mainly associated with industrialized countries, whereas it is now one of the most diagnosed tumors. Moreover, it is assumed that cases will rise in the near future as epidemiological data show a significant increase in cases in developing countries [2]. This implies a greater challenge for healthcare systems. Based on data from the International Agency for Research on Cancer (IARC), colorectal cancer (CRC) cases are expected to be 65% higher by 2040, and mortality will increase by 73% [2].

Leptin and adiponectin are peptide hormones produced predominantly by adipose tissues (AT), which exhibit opposing effects on tumor growth. Leptin promotes cancer development and metastasis, while adiponectin attenuates it. Altered levels of leptin and adiponectin, as well as imbalances in their production, disrupted assembly, secretion and signal transduction, are key determinants in malignancy [3]. The correlation of leptin and adiponectin with various cancers is well documented and includes both adenocarcinomas and squamous cell carcinomas [4,5]. According to Pu and Chen, both adipokines are implicated in neoplastic transformation and often show opposite actions, sharing common intracellular signaling pathways that contribute to cancer progression [6]. Hence, taken together, these two adipokines represent promising biomarker candidates in colorectal tumorigenesis, with potential value in cancer development and prognosis.

Notably, research provides controversial results regarding serum leptin and adiponectin levels in gastrointestinal malignancies. For example, a number of studies have reported contrasting leptin levels in patients with CRC. Some researchers report elevated leptin levels [7], while others show decreased levels [8]. There are even data reporting unaltered levels [9,10]. In contrast, adiponectin levels have been observed to either decrease [9,11] or remain unchanged [12]. These discrepancies may stem from the fact that malignant transformation is influenced not only by serum adipokine concentrations but also by the expression of adipokine receptors on various cells, which reflect tissue sensitivity [12]. Additionally, differences in findings may be attributed to the heterogeneity of study populations concerning disease stage [7], tumor location [9], sex [13], and weight changes [14].

In addition, data reported by Kim et al., 2024, show different activities of leptin and adiponectin in colorectal neoplasia [15]. Elevated leptin levels correlate with lymph node involvement, microvascular invasion, and advanced tumor stage, whereas decreased adiponectin levels are associated with suppression of colon cancer cells by adiponectin’s receptor-mediated AMPK activity [15].

In this review, we provide a comprehensive overview of current knowledge regarding the complex relationship between adipokines, leptin and adiponectin, and CRC. This study emphasizes the latest advances in the field of adipokines’ pathways and CRC pathogenesis and progression.

## 2. Colorectal Cancer Risk Factors

In CRC, there is a significant contribution of environmental factors, as most of the cases are sporadic and only a small percentage is associated with certain hereditary alterations [16]. The adenoma–carcinoma sequence, in which malignant tumors develop from a subset of adenomatous polyps over decades, is the main cause of sporadic CRC. Moreover, this type of malignancy is more common in populations where spontaneous adenomas are highly prevalent [17]. Furthermore, people who have a history of polyps are more likely to acquire CRC than people who do not. The malignant transformation’s underlying molecular pathways are largely recognized. Somatic mutations in proto-oncogenes and tumor-suppressor genes are among the progressive genetic abnormalities that are a part of this process [18,19,20,21,22].

Risk factors contributing to CRC include age, genetic predisposition, and lifestyle factors (https://www.who.int/news-room/fact-sheets/detail/colorectal-cancer (accessed on 19 April 2025)) [23]. Notably, recent data indicate an increasing prevalence of early-onset colorectal cancer (EOCRC) in adults under 50 [24]. This tendency is attributed in part to the Western diet, which directly affects the gut microbiota and indirectly modifies metabolic processes [25]. Gastrointestinal tract is the largest endocrine organ in the body, which expresses more than thirty hormone genes and numerous bioactive peptides known up to now [26].

### Obesity and Colorectal Malignancy

Epidemiological studies conducted over the past 30 years have consistently reported a positive association between obesity and colorectal neoplasia [27,28,29,30,31,32]. Adipose tissue participates as a central mediator of the inflammatory process, secreting hormones, growth factors and pro-inflammatory cytokines that are of particular importance in the pathogenesis of CRC [27]. Additionally, obesity-induced insulin resistance leads to elevated plasma insulin, glucose, and fatty acid levels. Exposure of colonocytes to elevated concentrations of insulin has mitogenic effects on these cells, whereas exposure to glucose and fatty acids can induce metabolic disturbances, changes in cell signaling pathways, and oxidative stress [27].

Obesity is a multifactor metabolic disorder that causes low-grade chronic inflammation and altered secretion of adipokines and cytokines, imbalanced insulin levels, aberrations of the insulin-like growth factor 1 (IGF-I) axis, changes in hormone biosynthesis and pathways, abnormal levels of adipokines and inflammation-related mediators such as IL-1β and TNF-α [33]. In addition, inflammation plays a key role in the development and progression of CRC [34]. Epidemiological evidence suggests that increased bowel inflammation is a major risk factor for the development of CRC [35]. Furthermore, experimental findings show that induced gut inflammation in mice leads to the development of colon cancer, indicating a direct link of inflammation to CRC [36].

In addition, dysregulation in adipokine synthesis and secretion, which has been reported in obesity, provides a favorable microenvironment for tumor development. Normally, adipokine production in adipose tissue is in balanced proportions. In state of chronic obesity that results in a condition of low-grade systemic inflammation, the production and secretion of the most abundant adipokines, leptin and adiponectin, are disrupted [37,38]. In obesity, excessive adiposity correlates with an increase in leptin levels and a decrease in adiponectin levels. Moreover, enlarged adipocytes in visceral adipose tissue (VAT) alter their secretions and acquire a pro-inflammatory phenotype [39]. This inflammatory state, associated with adipocytes, provides the secretion of significant amounts of pro-inflammatory factors, which in turn leads to alternation of key signaling messengers associated with tumor growth and progression [40].

Furthermore, a current large prospective cohort study among 44,271 Japanese men demonstrated strong mediating roles of circulating leptin and adiponectin to CRC risk [41]. In this research, Taguri and colleagues investigated the causal pathways linking obesity with colorectal malignancy with focus on plasma adipokines, biomarkers of chronic inflammation (CRP) and hyperinsulinemia (C-peptide). Advanced statistical analyses indicated that higher body mass index (BMI) is associated with an increased risk of CRC, mediated by leptin and adiponectin. Leptin, C-peptide and CRP showed positive correlation with CRC risk while adiponectin tends to possess protective effects. Their findings highlight the significant role of these two adipokines in the biological mechanisms that trigger CRC development.

## 3. Leptin Characteristics

Leptin is a 16 kDa peptide hormone encoded by the obesity gene (*ob* gene) on chromosome 7q31.3, which was first identified in 1994 [42]. Structural analysis shows that this adipokine is a member of the growth hormone four-helical cytokine subfamily, that includes ciliary neurotrophic factor, granulocyte colony-stimulating factors, growth hormone, erythropoietin, interleukins 2, 3, 4, 5, and 10, and leukemia inhibitory factor [43].

Leptin, known as an anti-obesity hormone, is a low molecular weight (167 amino acids) hormone that is synthesized and secreted predominantly by the white adipocytes [42]. Leptin is secreted to a greater extent from subcutaneous adipose tissue compared to visceral. Besides adipose tissue, it is expressed in other tissues and organs, such as stomach [44] and other parts of gastrointestinal system (https://www.proteinatlas.org/ENSG00000174697-LEP/tissue#rna_expression (accessed on 27 June 2025)). [45], and placenta [46]. Furthermore, leptin is expressed in some tumor tissues like breast cancer tissue [47] and CRC [48]. Interestingly, in colorectal malignancy, this adipokine is significantly overexpressed in more aggressive tumors (like highly undifferentiated tumors).

The synthesis and secretion of leptin are proportional to both the number and size of adipocytes [49]. Moreover, this adipokine is secreted to a greater extent from subcutaneous adipose tissue compared to visceral. Circulating leptin levels in healthy individuals with normal body weight typically range from 5 ng/mL to 15 ng/mL, while in obese individuals, these levels can reach 100 ng/mL and exceed 250 ng/mL [5]. In addition, physiological leptin concentrations are inversely related to circadian rhythm. They are lowest in the morning, as well as early in the evening, and show elevations at night [5].

In the bloodstream, this adipokine is found in free form or bound to its soluble receptor. The ratio of the free to bound form shows individual variability depending on the white adipose tissue content of the body. In obese individuals, leptin is mainly presented in its free form, while in lean individuals, it is primarily found in the protein-bound form [50]. It should be noted that serum leptin levels are higher in women than in men, even when adjusted for age and BMI [51]. This is related to the greater amount of subcutaneous fat, which secretes leptin more intensely relative to visceral fat in women, and the influence of sex hormones. Estrogens stimulate increased leptin release from adipocytes, whereas testosterone shows an inhibitory effect [52].

### 3.1. Biological Activities of Leptin

Leptin exerts pleiotropic activities, affecting a diverse range of physiological processes throughout the body. After active transport through the blood–brain barrier (BBB), it binds to specific receptors that are located in the hypothalamus and leads to the release of appetite-suppressing neuropeptides, i.e., it represents a satiety signal and has a key role in appetite regulation and energy homeostasis. In addition to the regulation of body mass and energy expenditure, leptin is involved in the regulation of hematopoiesis [53], immune response [54], angiogenesis [55], control of blood pressure [56], bone formation [57] and reproduction [57]. It is worth mentioning that, in humans, the deficiency of this hormone or resistance to its physiological effect results in the development of obesity, diabetes, and infertility [58]. Some authors have suggested that obesity may result from leptin resistance when obesity increases and leptin levels become chronically elevated [59]. Leptin resistance in humans is increasingly reported in modern societies where energy-rich food is widely available. In high-fat diet-induced obesity, despite an increase in circulating leptin levels, the BBB permeability to leptin decreases. This disrupted leptin transport through the BBB is one of the reasons for leptin resistance [60]. Moreover, induced hyperleptinemia in obese individuals causes hyperglycemia and hyperinsulinemia that lead to diabetes mellitus.

Moreover, leptin participates directly in inflammation by increasing phagocytic activity of macrophages and production of C-reactive protein (CRP) [61]. In addition, the synthesis of leptin is increased by insulin, pro-inflammatory factors such as TNF-alpha, IL-6, by glucocorticoids and estrogens, and suppressed by androgens, free fatty acids, and growth hormones.

### 3.2. Leptin Receptors and Signaling Pathways in Colorectal Cancer

Leptin exerts its activities in various organs that express the leptin receptor, such as the brain, adipose tissue, gastrointestinal tract, muscles, or kidneys, with its main effect on hypothalamic regions [57]. Leptin receptors (LepRs, ObRs) exist in six isoforms with different lengths and C-terminal sequences. These isoforms are products of alternative RNA splicing of the LEPR gene. According to structural differences, the receptor isoforms are divided into three classes: long (LepRb), short (LepRa, LepRc, LepRd and LepRf) and secretory isoforms (LepRe) [62]. Leptin receptors, both long and short isoforms, can bind to Janus-activated kinases (JAKKinase) and transduce several intracellular signaling pathways [63]. Leptin induces two key cell growth signaling pathways in CRC. The first pathway of leptin, after binding to its receptor type JAKKinase, leads to its phosphorylation, followed by binding and activation of STAT, which dimerizes and translocates into the nucleus. This results in upregulation of the expression of various STAT3-responsive target genes that mediate the primary actions of leptin. One of these is the suppressor of cytokine signaling SOCS3. This molecule acts as a potent negative regulator of the JAK/STAT signaling pathway. This mechanism of inhibition by SOCS3 appears to be related to the leptin receptor, as its mutation prevents the feedback mechanism [64]. Increased SOCS3 activation, which in turn would inhibit the JAK2-STAT3 signaling pathway, is one of the major mechanisms involved in leptin resistance [65]. Leptin also activates the PI3K enzyme, leading to phosphorylation and activation of Akt (Protein Kinase B) to induce an intracellular signaling cascade [66]. Akt stimulates downstream targets, including mTOR, which promotes protein synthesis, cell growth, and survival. This pathway is critical in angiogenesis, glucose metabolism, and tumor growth [67]. In addition, recent study indicated the role of leptin and its receptors in obesity-associated tumors via the activation of these pathways [68]. For example, in colon tumors, leptin-stimulated cell proliferation and survival are associated with the ObRL/STAT3 signaling pathway. Moreover, even in severe obesity cancer, proliferation was reported to be inhibited in case of deficiency of leptin and its receptor [68].

Leptin receptor overexpression in CRC patients is indicative for active leptin-mediated communication [69]. Moreover, leptin has stimulating effect on DNA replication and growth of CRC cells and, when its expression is upregulated, it serves as a growth factor via the MAPK and PI3-K pathways [48,70]. Furthermore, in a review published in 2022, Socol and colleagues reported reduction in tumor proliferation and metastasis in the absence of leptin receptors in CRC patients’ tissues in most of the cases [71].

Interestingly, data on the role of the soluble leptin receptor in CRC pathogenesis is still insufficient. In a wide range prospective case–control study, Aleksandrova et al. showed a strong negative correlation between circulating levels of LepRe and risk of colon cancer [72]. These findings suggest that probably high circulating levels of soluble leptin receptor may possess a protective role in CRC. Additionally, they reported that leptin alone was not significantly associated with CRC risk.

## 4. Adiponectin Characteristics

Adiponectin is a hormone synthesized predominantly by adipose tissue (AT). It is produced mainly by mature adipocytes, with subcutaneous adipose tissue secreting more adiponectin than visceral tissue [73]. In addition to AT, adiponectin production is known to occur in bone [74], placenta [75], cardiomyocytes [76], pituitary gland [77], skeletal muscle [78] and digestive tract [79]. Adiponectin is encoded by the AdipoQ gene, and its monomer is a molecule composed of 244 amino acids that consists of a signaling region at the NH2-terminus, a variable region, a collagen-like domain, and a globular domain at the COOH-terminus [80]. Adiponectin is secreted as a monomer that forms complexes of different molecular weights circulating in plasma [81]. These complexes can be trimeric (LMW), hexameric and multimeric (12–18) molecules [82]. Circulating adiponectin is mainly a medium molecular weight form (MMW, hexamer, approximately 180 kDa) and a high molecular weight form (HMW, dodecamer or octadecamer, approximately 360 or 540 kDa) [83].

Adiponectin is the most abundant adipokine produced by white adipocytes. In normal physiological conditions, this adipokine represents about 0.01% of total serum protein in concentrations of 5–50 µg/mL. Interestingly, serum adiponectin levels are inversely proportional to BMI and insulin resistance. In pathological conditions caused by chronic inflammatory processes, adiponectin levels decrease [84]. Reported levels in women are almost twice as high as in men, as androgens have an inhibitory effect on adiponectin gene expression. Therefore, sex-related differences in circulating blood adiponectin levels are observed [85]. Similar to leptin, circulating adiponectin levels show diurnal fluctuations. In this sense, adiponectin reaches its maximum expression early in the morning, and its concentration significantly decreases late in the evening [86].

### 4.1. Biological Activities of Adiponectin

Adiponectin exerts pleiotropic effects on various tissues and organs. Of interest is the fact that different isoforms of adiponectin mediate different effects in different tissues and organs. For example, the high molecular weight multimeric isoform is biologically the most active and is thought to mediate the pro-inflammatory effects of adiponectin, while the trimeric isoform is presumed to be responsible for its anti-inflammatory activity [87]. Adiponectin possesses insulin-sensitizing [88] and antiatherogenic [89] properties. The main role of adiponectin is in maintaining homeostasis in carbohydrate and lipid metabolism. This adipokine enhances fatty acid oxidation in both skeletal and cardiac muscle, as well as in the liver, thereby reducing triglyceride content in these tissues [90]. In addition, it stimulates glucose uptake by skeletal and cardiac muscle and inhibits glucose production by the liver, resulting in lower blood sugar levels.

Various studies have indicated that low circulating adiponectin levels are uniquely associated with obesity, insulin resistance and type 2 diabetes [91,92,93]. Moreover, insulin possesses direct mitogenic effect on colonocytes confirmed in both in vivo and in vitro experiments [94,95]. In addition, Griffen and colleagues reported that increased insulin levels can exert both growth-promoting and metabolic effects [96]. The hyperinsulinemia detected in insulin resistance is hypothesized to enhance cellular proliferation via the mitogen-activated protein (MAP) kinase pathway [97] and to decrease apoptosis through the phosphatidylinositol 3-kinase and Akt pathway [98]. 

In the context of cancer, adiponectin has been shown to inhibit cell proliferation and induce apoptosis, suggesting a protective role against tumorigenesis [99].

Angiogenesis is central element in cancer development and metastasis. Notably, regarding published reports, the effect of adiponectin on tumor neovascularization remains controversial. For example, some studies show proangiogenic characteristics of adiponectin [100], while others report antiangiogenic properties [101,102].

### 4.2. Adiponectinn Receptors and Signaling Pathways in Colorectal Cancer

The pleiotropic activities of adiponectin are mediated via two canonical receptors, AdipoR1 and AdipoR2, and one non canonical T-cadherin. The two classical receptors trigger two different signaling pathways. AdipoR1 activates an AMP-dependent kinase, while AdipoR2 activates the PPARα cascade [90]. AMP-dependent kinase is an enzyme that responds to a low ATP/AMP ratio, a signal of a low-energy state of the cell. The activation of AMP-dependent kinase stimulates processes that aim to increase ATP-increased glucose transport and fatty acid oxidation while inhibiting fatty acid, protein and glycogen synthesis [103]. When the adiponectin signal transduction pathway is functional, activation of this enzyme provokes a shift from energy storage to ATP production. Thus, to maintain energy homeostasis, AMPK inhibits ATP-demanding anabolic pathways, including those supporting cell growth and proliferation. In addition, activation of the AMPK signaling pathway suppresses the expression of the myeloid leukemia cell differentiation protein (mcl-1 protein) by targeting mTOR [104]. The Mcl-1 protein is a well-known inhibitor of apoptosis in the cancer cells controlling early stages in the programmed cell death cascade [105]. Notably, AMPK is also a key negative regulator of the Warburg effect, a well-known metabolic hallmark of cancer cells [106]. Furthermore, evidence indicates that adiponectin inhibits malignant cell growth in CRC by activating AMPK signaling and subsequent suppression of the mTOR pathway [107].

Adiponectin also activates PPARα (peroxisome proliferator-activated receptor α), the first discovered subtype of transcriptional factors belonging to the nuclear hormone receptor superfamily [108]. PPARs are key regulatory proteins that maintain energy homeostasis by modulating lipid and glucose metabolic pathways. Along with metabolic regulation, PPAR-activated signaling cascades participate in the coordination of cellular processes such as proliferation, differentiation, and apoptosis [109]. Due to this broad regulatory role, disturbances in PPARs signaling are associated with the development of metabolic, inflammatory, and immune pathologies, as well as various forms of tumorigenesis [110]. It has recently been established that human colorectal tumors demonstrate significantly reduced expression of PPARα mRNA and protein compared to normal intestinal tissues. Additionally, the activation of intestinal PPARα provides a protective effect against colorectal carcinogenesis [111]. Moreover, a recent study reported that the loss of PPARα stimulates CRC tumor immune escape [112]. This data emphasizes that factors capable of modulating PPAR-mediated signaling pathways in the colon represent promising therapeutic and prophylactic strategies for the prevention and control of colorectal carcinoma.

Intriguing, AdipoR2 is now known to possess ceramidase activity, which is enhanced by adiponectin binding [113]. The specific function of adiponectin to induce ceramidase activation results in decreased hepatic ceramide levels and promotes their catabolism. Elevated adiponectin receptor levels lead to reduced ceramide levels and improved total body glucose and lipid homeostasis by an adiponectin-dependent mechanism [114]. High intracellular ceramide levels significantly compromise insulin signaling, thereby predisposing the liver to the onset of insulin resistance and the development of type II diabetes [115]. The beneficial effects of adiponectin, particularly its insulin-sensitizing effect, are likely closely related to the ceramidase activity of its own receptors. Although the mechanism is not yet clear, some studies suggest that adiponectin directly opposes the damaging effects of TNFα in liver tissue [83].

T-cadherin is a receptor for HMW adiponectin but not for trimeric or globular adiponectin [116]. In addition, Kita et al., 2019, reported that native adiponectin in serum binds to mammalian cells expressing T-cadherin, but not AdipoRs, demonstrating that T-cadherin is a major receptor for native adiponectin [117].

It has become evident that T-cadherin expression is commonly suppressed by abnormal methylation in colorectal cancer and adenomas. Moreover, in 2004, Hibi et al. demonstrated that, in the poorly differentiated CRC, CDH13 gene that encodes the synthesis of T-cadherin, methylation is frequently compared to that in differentiated ones [118]. Since CDH13 encodes a cell adhesion protein, its inactivation through promoter methylation contributes to the loss of cell cohesion seen in poorly differentiated tumors. Intriguing, T-cadherin functions as a receptor for two ligands—adiponectin and low-density lipoprotein (LDL)—that are competing to bind with this receptor in health and disease [119]. In obese individuals’ serum, adiponectin levels are significantly reduced and T-cadherin becomes occupied by LDL.

T-cadherin expression is reduced in numerous cancers such as ovarian, endometrial, breast, lung, gallbladder carcinomas, pituitary adenomas, malignant B cell lymphomas, nasopharyngeal carcinoma, osteosarcoma and colorectal carcinomas [120]. That is why it was proposed that T-cadherin serves as tumor suppressor, but the exact molecular mechanisms are still not completely understood.

Abundant epidemiological data showed that decreased adiponectin levels are oppositely associated with increased risk of CRC, especially in men [11,121,122]. Moreover, in vitro studies with cell lines additionally contribute to these findings. Adiponectin application has been reported to stimulate apoptosis, reduce proliferation and control the expression of cell cycle genes by phosphorylation of AMPK [123]. Also, Moon et al., 2013, demonstrated that adiponectin provides direct regulation of malignant determinants (cell proliferation, adhesion and invasion) and controls metabolic (AMPK/S6), inflammatory (STAT3/VEGF) and cell cycle pathways (p21/p27/p53/cyclins) in several human cell lines as well as in vivo in a mouse model [124]. Furthermore, it has been shown that hypo-adiponectinaemia additionally enhances CRC development and suppress G1/S cell cycle arrest by regulating AMPK and mTOR-related signaling pathways [125].

**Figure 1:**
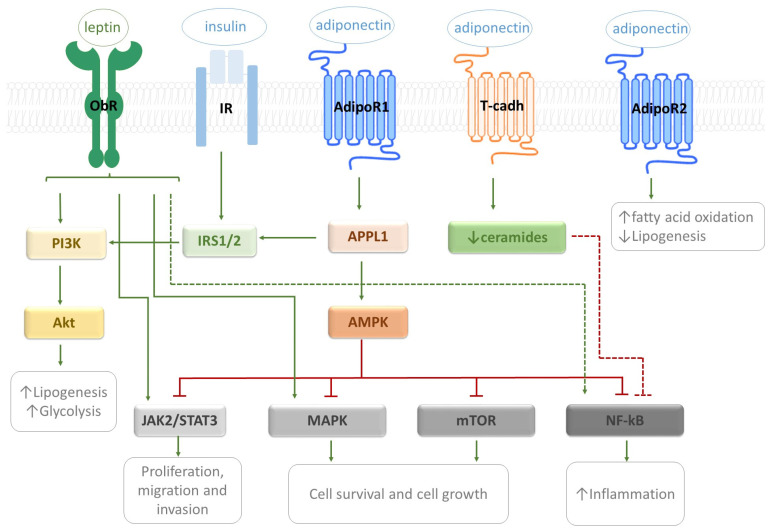
Counteractive interaction between leptin and adiponectin signaling in CRC/. Solid lines represent direct interactions (green arrows for activation and blunt-end red lines for inhibition), whereas dashed lines represent indirect effects. Leptin and adiponectin trigger signaling factors that integrate PI3K/Akt, MAPK, mTor, and NF-kB pathways. Leptin stimulation of ObR leads to PI3K/Akt activation and subsequent promotion of lipogenesis and glycolysis. Additionally, leptin receptor stimulation activates JAK2/STAT3 cascade resulting in increased proliferation, migration and invasion of cancer cells. The activation of ObR supports the MAPK and NF-kB activation which reflects pro-proliferative signaling and inflammation. Adiponectin acts through several receptors like AdipoR1, AdipoR2 and T-cadherin. Adiponectin stimulation of AdipoR1 leads to AMPK activation with the help of adaptor protein APPL1. AMPK activity results in the inhibition of MAPK, mTOR, and NF-κB signaling pathways. Therefore, adiponectin suppresses cell growth and inflammatory responses. Meanwhile, the engagement of AdipoR2 leads to the stimulation of fatty acid oxidation and repression of lipogenesis. In addition, the activation of T-cadherin by adiponectin decreases intracellular ceramide levels. The reduction in ceramide levels contributes to lower inflammation.

Complementing the interconnected activities of leptin and adiponectin in CRC, Figure 1 represents major signaling pathways in CRC modulated by these adipokines.

## 5. Leptin and Adiponectin as Dual Modulators of Cancer Metabolic Remodeling

The tumor metabolic reprogramming driven by adipokines is recognized as crucial determinant in obesity-associated cancers [3]. The first identified metabolic phenotype of cancer cells is the aerobic glycolysis, a phenomenon named the “Warburg” effect. Nowadays, it is known that the Warburg effect is a process programmed by numerous oncogenes. For instance, an increase in glucose uptake and the resulting upregulation of glycolytic flow is driven by the overexpression of the glucose transporters (GLUTs) [126]. Moreover, intermediate components of glycolysis can be redirected to alternative pathways that provide the link between glycolysis and other metabolic pathways, such as the biosynthesis of phospholipids, pentose phosphate, hexosamine and amino acids [127]. Consequently, malignant cells develop proliferative and biosynthetic advantages compared to non-malignant cells driven by the increased glycolytic flux.

It has become evident that leptin intensifies the Warburg effect through different mechanisms. Abundant data reported in experiments with malignant and non-malignant cells show that leptin induces glucose utilization by regulating GLUT-1 [128,129]. Furthermore, leptin stimulates glycolysis through regulating the activity and expression of key enzymes in glycolytic pathways, like hexokinase, lactate dehydrogenase A (LDHA) and pyruvate kinase M2 (PKM2) [128,130].

**Figure 2:**
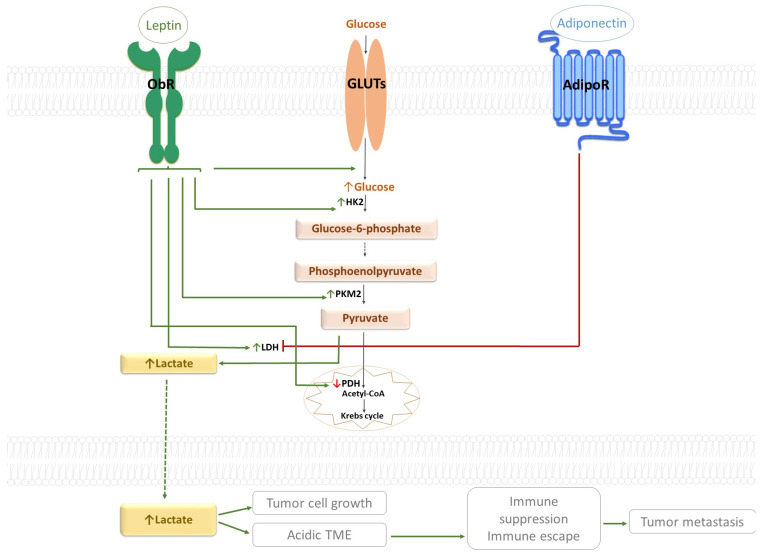
The opposite effects of leptin and adiponectin on the Warburg effect. Solid lines represent direct interactions (green arrows for activation and blunt-end red lines for inhibition), whereas dashed lines represent indirect effects. Leptin stimulation of ObR enhances glucose uptake through the activation of GLUTs. Leptin signaling also upregulates key glycolytic enzymes like hexokinase 2 (HK2), and pyruvate kinases M2 (PKM2) and thus promoting glucose to pyruvate conversion. Subsequently, the leptin activation leads to the reduction of pyruvate to lactate by upregulation of lactate dehydrogenase (LDH). Elevated lactate levels contribute to cancer cell growth, extracellular acidification and immune escape. Moreover, leptin stimulation downregulates pyruvate dehydrogenase (PDH) and thereby disturbs pyruvate influx into the Krebs cycle. This additionally increases the pyruvate to lactate conversion resulting in increased lactate levels. In contrast, the engagement of AdipoR by adiponectin has inhibitory effect on LDH and promoting pyruvate entry into the Krebs cycle. These dynamic interactions illustrate the opposing roles of leptin and adiponectin on Warburg effect.

In addition to leptin, adiponectin also possesses well-known effects in glucose metabolism. It has become evident that under normal physiological conditions, adiponectin facilitates glucose uptake and utilization because of stimulation of insulin signaling [131]. Increased glucose uptake induced by adiponectin is provided via the upregulation of GLUT-4 translocation in skeletal muscles [132]. However, it is still not yet verified the exact effect of this adipokine on glucose metabolism in cancer cells. Nevertheless, current data reports that adiponectin induces oxidative phosphorylation of glucose while suppressing glycolysis and lactate production, a metabolic change that contrasts with the Warburg effect [133]. The opposite effects of leptin and adiponectin on the Warburg effect are illustrated in Figure 2.

## 6. Antagonistic Effect of Adiponectin Toward Leptin in Tumors

It is worth mentioning that adiponectin antagonizes the effect of leptin on tumor growth [68,134]. It has been reported in hepatocellular carcinomas [135], breast cancer [136], and endometrial cancer [137]. A study conducted by Fenton et al., 2008 demonstrated the inhibitory effect of adiponectin to multiple signaling pathways associated with leptin-induced cell proliferation in Apc Min/+ colon epithelial cells (a model of preneoplastic colon cells) [138]. These data were supported by Yamaji et al. [139]. In 2010, they reported the first epidemiological evidence for cooperative effects of adiponectin and leptin in the early-stage CRC, hypothesizing that adiponectin may possess an antitumor activity on the colon by interfering with leptin, and leptin may express a pro tumorigenic effect in case of decreased levels of adiponectin.

## 7. Leptin, Adiponectin and Metastasis Process in CRC

Leptin activates several critical signaling pathways in cancer cells, which triggers metastasis process in colorectal malignancy. As was mentioned previously, this adipokine directly stimulates the JAK/STAT3 signaling cascade associated with cell growth and tumor metastasis, possessing a key role in the metastatic potential of CRC cells. Moreover, it is a critical effector of PI3K/AKT/mTOR signaling and also a strong regulator of cancer cell proliferation. In addition, the activation of PI3K/AKT/mTOR pathway by leptin is related to inhibition of cancer cell apoptosis, enhancing survivability of the tumor cells. Recent research, involving 407 CRC patients, has identified leptin as an independent risk factor for metastatic colorectal cancer [48]. Li and colleagues have reported significant correlation between strong leptin expression in CRC tissues and the presence of lymph node and liver metastases. Furthermore, tissue expression of this adipokine progressively increased from the early to the advanced stages of the malignant process.

Regarding the expression of leptin receptors in CRC patients, Parmesh et al. have demonstrated that LEPR is a stronger indicator of tumor progression than leptin [140]. Additionally, they reported markedly decreased expression of ADIPOR1 in distant metastases. Their findings postulated that ADIPOR1 has better prognostic significance in advanced CRC than ADIPOR2 and ADIPOQ. Intriguingly, another study from the same author postulated that positive expression of leptin and negative expression of adiponectin receptors in CRC could be a strong predictive marker for metastatic risk [141].

Most experimental studies have shown that adiponectin possesses antiproliferative activities in cancer process [142]. Through the activation of the AMPK signaling pathway and inhibition of STAT3, this adipokine reduces migration and invasion of CRC cells and thus suppresses metastatic behavior. Besides this, it is important to note that some studies indicate proliferative activities of adiponectin in CRC [143]. This data underlines the bifurcate role of adiponectin in the malignant process depending on cellular context [144].

## 8. Role of Leptin and Adiponectin in Chemoresistance and Treatment Response in CRC

Chemoresistance in cancer is a serious challenge in clinical practice associated with poor outcomes. The effectiveness of chemotherapy is often compromised by the development of drug resistance in cancer cells. The role of both adipokines, leptin and adiponectin, in this process is still under investigation. Recent scientific evidence indicates that leptin plays a context-dependent role in chemoresistance in CRC. In obesity-associated disease, elevated leptin level has been shown to promote resistance to commonly used chemotherapeutic agent 5-fluorouracil [145]. This is due to the suppression of AMPK activity and upregulation of drug efflux mechanisms. In contrast, a recent experimental study reported that leptin enhances chemosensitivity to a certain agent, like cisplatin [146]. Together, these findings highlight the dual and highly specific effects of leptin on colorectal cancer therapy response.

To our knowledge, no data on the effect of adiponectin on chemoresistance in CRC have been published to date.

## 9. Adiponectin Receptor Agonists and Leptin Antagonists as Potential Candidates in CRC Treatment

An intriguing therapeutic target in CRC is adiponectin signaling characterized by its well-known tumor-suppressive effects. Synthetic adiponectin receptor agonists, mainly AdipoRon, are being explored as anticancer agents [147]. Recent data in CRC are still preclinical. AdipoRon activates AMPK and inhibits mTOR signaling pathways, significantly reducing proliferation and colony formation in several CRC cell lines, like HCT116, HT29, LoVo [148,149]. Furthermore, treated CRC cells demonstrated an increased predisposition to apoptosis. Similar results are reported in studies with colon organoids [150]. AdipoRon lowers plasma-membrane cholesterol and thus it suppresses canonical Wnt-signaling. In addition, it reduces Lgr5+ stem cells in colon organoids, associated with stem-cell-driven tumorigenesis.

Leptin signaling could be a key target for new therapeutic interventions in colorectal malignancy. Leptin signaling inhibitors targeting critical oncogenic pathways demonstrated considerable potential in preclinical cancer trials [151]. Recent studies have revealed the existence of a complex communication axis in malignant diseases called NILCO, specific crosstalk between Notch, IL-1, and leptin signaling [152,153]. It represents a complex signaling interaction that is the main driver of the oncogenic effects caused by leptin. Leptin can activate Notch and IL-1, which in turn can enhance the effects of leptin. This interaction generates pro-inflammatory and proangiogenic signals that promote tumor growth and invasion. Consistent with these findings, in 2023, Özyurt and colleagues reported that blocking the NILCO signaling axis through Allo aca, an ObR antagonist, causing reduction in proangiogenic factors, like VEGF and provoking apoptosis in xenograft colorectal cancer [154].

## 10. Conclusions

Over the past 20 years, CRC has gained considerable attention, probably due to the rapidly increasing number of reported cases, especially in obese individuals and notably in younger people. There is unfortunately an increasing incidence of CRC in the young generation. In addition, in a large prospective study among Norwegian adolescents has been reported a twofold higher risk of mortality in adulthood among overweight teenagers with CRC [155]. All these findings indicate that CRC remains a major health challenge, with growing rates strongly related to lifestyle and metabolic aspects, involving obesity.

The complex involvement of obesity-associated adipokines, in particular leptin and adiponectin, has been investigated in numerous comprehensive studies over the past decade. The leptin’s oncogenic role in colorectal malignancies has been strongly underlined by current data, primarily by activation of the JAK2/STAT3 and PI3K/Akt/mTOR signaling pathways. In contrast, adiponectin has been shown to act predominantly as a tumor suppressor in CRC because of the activation of AMPK and PPARα signaling cascades.

Despite significant progress that has been made in elucidating the signaling networks of these two adipokines in colorectal malignancy, challenges remain and a lot of questions await answers. The areas that require further investigation are crosstalk between leptin and adiponectin signaling, regulation by other adipokines, and post translational modifications of their receptors in tumor tissues. In addition, polymorphisms in LEP, LEPR, ADIPOQ, and ADIPOR genes may contribute to individual susceptibility and could represent potential molecular markers for CRC. Further elucidation of the role of leptin and adiponectin may contribute to the development of more precise and effective strategies for the prevention, early diagnosis, and treatment of colorectal cancer.

## Data Availability

No new data were created or analyzed in this study. Data sharing is not applicable to this article.

## Abbreviations

The following abbreviations are used in this manuscript:

CRC	Colorectal cancer
IARC	International Agency for Research on Cancer
AT	Adipose tissue
EOCRC	Early-onset colorectal cancer
IGF-I	Insulin-like growth factor 1
IL-1β	Interleukin 1 beta
TNF-α	Tumor necrosis factor alpha
CRP	C-reactive protein
BMI	Body mass index
BBB	Blood–brain barrier
LepRs/ObRs	Leptin receptors
LepRb/LepRa/LepRc/LepRd/LepRf/LepRe	Leptin receptor isoforms
JAKKinase	Janus-activated kinase
STAT/STAT3	Signal transducer and activator of transcription 3
SOCS3	Suppressor of cytokine signaling 3
PI3K	Phosphoinositide 3-kinase
Akt	Protein kinase B
mTOR	Mechanistic target of rapamycin
MAPK	Mitogen-activated protein kinase
VAT	Visceral adipose tissue
LMW	Low molecular weight
MMW	Medium molecular weight
HMW	High molecular weight
AdipoR1/AdipoR2	Adiponectin receptor 1 / 2
AMPK	AMP-dependent kinase
PPARα	Peroxisome proliferator-activated receptor alpha
mcl-1	Myeloid cell leukemia 1 protein
CDH13	Cadherin 13 gene
LDL	Low-density lipoprotein
VEGF	Vascular endothelial growth factor
GLUTs	Glucose transporters (general family)
GLUT-1	Glucose transporter 1
GLUT-4	Glucose transporter 4
LDHA	Lactate dehydrogenase A
PKM2	Pyruvate kinase M2
HK2	Hexokinase 2
LDH	Lactate dehydrogenase
PDH	Pyruvate dehydrogenase
Apc Min/+	Adenomatous polyposis coli, multiple intestinal neoplasia model (mouse model)
Lgr5+	Leucine-rich repeat-containing G-protein coupled receptor 5 positive stem cells
NILCO	Notch, IL-1, and leptin crosstalk signaling axis
